# Supplementary material for: Excessive ammonium assimilation by plastidic glutamine synthetase causes ammonium toxicity in Arabidopsis thaliana
Source: Nat Commun. 2021 Aug 16;12:4944. doi: 10.1038/s41467-021-25238-7 (PMC8367978; doi:10.1038/s41467-021-25238-7)
Supplement: Supplementary file 1 — Supplementary Information [file 41467_2021_25238_MOESM1_ESM.pdf]

Supplementary Information

Excessive ammonium assimilation by plastidic glutamine synthetase causes ammonium toxicity in *Arabidopsis thaliana*

Takushi Hachiya*, Jun Inaba, Mayumi Wakazaki, Mayuko Sato, Kiminori Toyooka, Atsuko Miyagi, Maki Kawai-Yamada, Daisuke Sugiura, Tsuyoshi Nakagawa, Takatoshi Kiba, Alain Gojon, & Hitoshi Sakakibara

Nature Communications (2021)

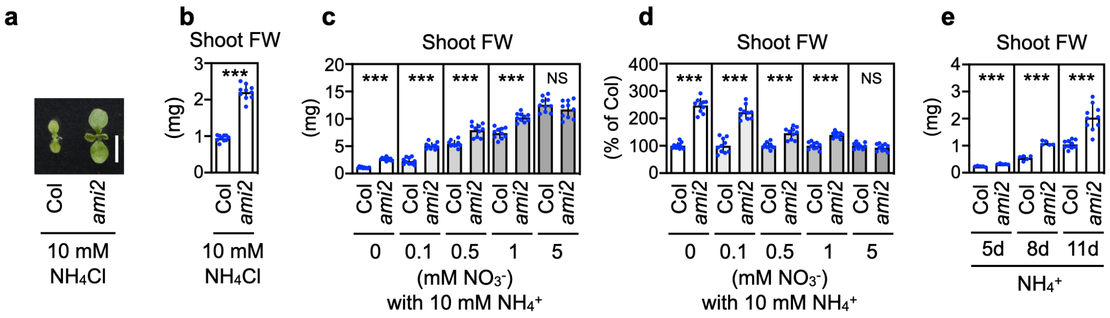

Supplementary Figure 1 The *ami2* mutant exhibits enhanced ammonium tolerance during early stages of development. **a** A representative photograph of shoots from the 11-d-old wild-type (Col) and *ami2* grown on media containing 10 mM NH_4Cl. **b** Fresh weights (FW) of shoots from 11-d-old Col and *ami2* grown on media containing 10 mM NH_4Cl (mean ± SD; n = 10). **c** FW of shoots and **d** relative FW (relative to Col as one) from 11-d-old Col and *ami2* grown on media containing 10 mM ammonium with varying concentrations of nitrate (mean ± SD; n = 10). **e** FW of shoots from 5- (mean ± SD; n = 5), 8- (mean ± SD; n = 5), or 11-d-old (mean ± SD; n = 10) Col and *ami2* grown on media containing 10 mM ammonium. **b-e** **P* < 0.05; ***P* < 0.01; ****P* < 0.001 (Welch's *t*-test). NS denotes not significant. Six shoots from one plate constituted a single biological replicate.

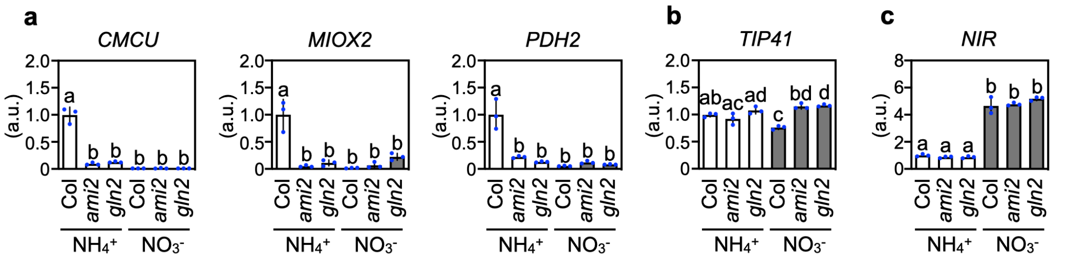

Supplementary Figure 2 Ammonium-dependent induction of ammonium-inducible genes are attenuated by *GLN2* deficiency. Relative transcript levels of **a** ammonium-inducible genes[9,23], **b** a house-keeping gene *TIP41*, and **c** nitrate-inducible gene *NIR*[26] in the shoots of 10-d-old Col, *ami2*, and *gln2* 3 d after transfer to media containing 10 mM ammonium or 10 mM nitrate (mean ± SD; n = 3). Six shoots from two plates constituted a single biological replicate. Different letters denote significant differences at $P < 0.05$ (Tukey-Kramer's multiple comparison test).

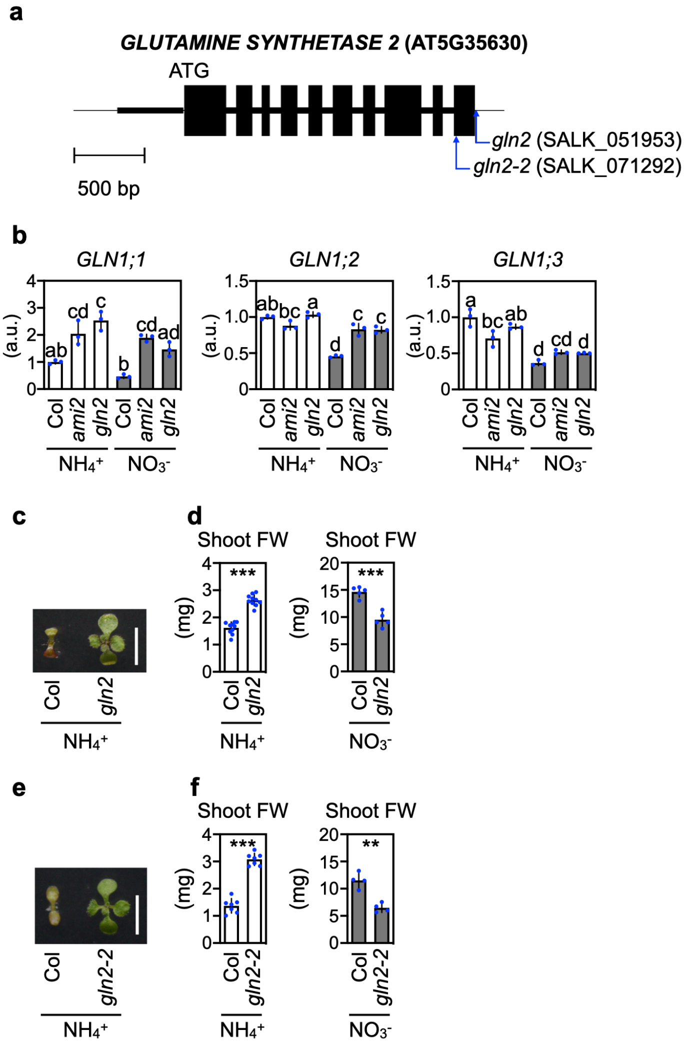

Supplementary Figure 3 ***GLN2* is a causative gene for ammonium toxicity. a** Genomic structure of the *GLN2* gene (AT5G35630). Black boxes, bold lines, and thin lines indicate exons, introns, and UTRs, respectively. The arrows denote the T-DNA insertion loci in *gln2* (SALK_051953) and *gln2-2* (SALK_071292). **b** Relative transcript levels of the *GLN1* genes in the shoots of 10-d-old Col, *ami2*, and *gln2* 3 d after transfer to media containing 10 mM ammonium or 10 mM nitrate (mean ± SD; n = 3). Six shoots from two plates constituted a single biological replicate. Different letters denote significant differences at $P < 0.05$ (Tukey-Kramer's multiple comparison test). **c** A representative photograph of shoots from the 11-d-old Col and *gln2* grown on media containing 10 mM ammonium. **d** Fresh weights (FW) of shoots from 11-d-old Col and *gln2* grown on media containing 10 mM ammonium (mean ± SD; n = 10) or 10 mM nitrate (mean ± SD; n = 5). **e** A representative photograph of shoots from the 11-d-old Col and *gln2-2* grown on media containing 10 mM ammonium. **f** FW of shoots from 11-d-old Col and *gln2-2* grown on media containing 10 mM ammonium (mean ± SD; n = 7) or 10 mM nitrate (mean ± SD; n = 4). **d,f** *$P < 0.05$; **$P < 0.01$; ***$P < 0.001$ (Welch's *t*-test). Six shoots from one plate constituted a single biological replicate.

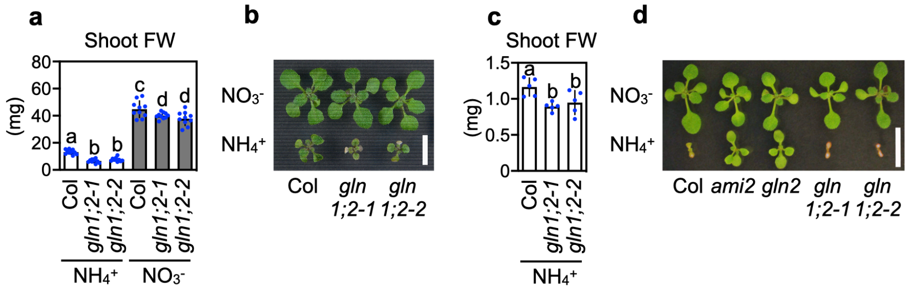

Supplementary Figure 4 A deficiency in GLN1;2 causes ammonium hypersensitivity. a FW of shoots from 14-d-old Col, *gln1;2-1*, and *gln1;2-2* 7 d after transfer to media containing 10 mM ammonium or 10 mM nitrate (mean ± SD; n = 10). One shoot from one plate constituted a single biological replicate. **b** A representative photograph of 14-d-old Col, *gln1;2-1*, and *gln1;2-2* 7 d after transfer to media containing 10 mM ammonium or 10 mM nitrate is shown. The scale bar represents 10 mm. **c** Fresh weights (FW) of shoots from 11-d-old Col, *gln1;2-1*, and *gln1;2-2* grown on media containing 10 mM ammonium (mean ± SD; n = 5). Six shoots from one plate constituted a single biological replicate. **d** A representative photograph of shoots from the 14-d-old Col, *ami2*, *gln2*, *gln1;2-1*, and *gln1;2-2* grown on media containing 10 mM ammonium or 10 mM nitrate. **a,c** Different letters denote significant differences at *P* < 0.05 (Tukey-Kramer's multiple comparison test).

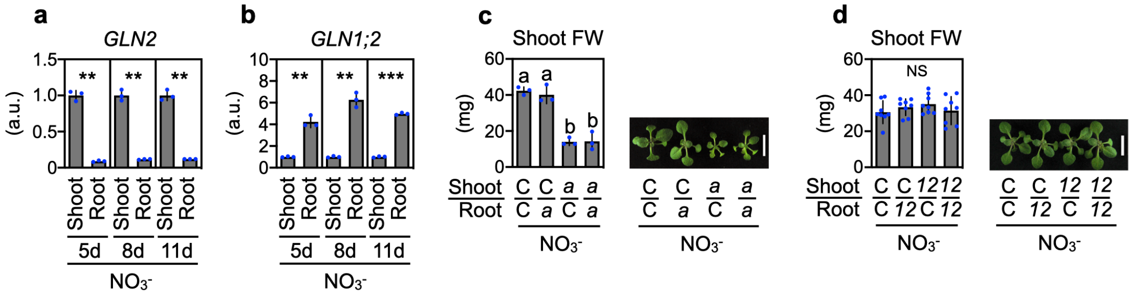

Supplementary Figure 5 Shoot *GLN2* is crucial for shoot growth when nitrate is the N-source. a Relative transcript levels of *GLN2* in the shoots and roots of 5-, 8-, or 11-d-old Col grown on 10 mM nitrate (mean ± SD; n = 3). **b** Relative transcript levels of *GLN1;2* in the shoots and roots of 5-, 8-, or 11-d-old Col grown on media containing 10 mM nitrate (mean ± SD; n = 3). **a,b** Twelve shoots and roots from one plate constituted a single biological replicate. *$P < 0.05$; **$P < 0.01$; ***$P < 0.001$ (Welch's *t*-test). **c** FW of shoots from 17-d-old reciprocally-grafted plants between Col (C) and *ami2* (a) 7 d after transfer to media containing 10 mM nitrate (mean ± SD; n = 3). **d** FW of shoots from 17-d-old reciprocally-grafted plants between Col (C) and *gln1.2-1*[12] 7 d after transfer to media containing 10 mM nitrate (mean ± SD; n = 8). **c,d** One shoot from one plate constituted a single biological replicate. Representative photograph of 17-d-old shoots 7 d after transfer to media containing 10 mM nitrate are shown. Different letters denote significant differences at $P < 0.05$ (Tukey-Kramer's multiple comparison test). NS denotes not significant. The scale bar represents 10 mm.

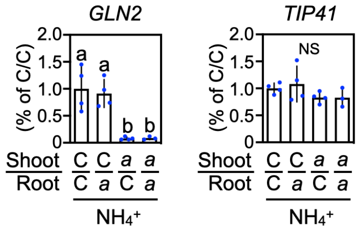

Supplementary Figure 6 Root expression of *GLN2* does not affect the transcript levels of shoot *GLN2*. Relative transcript levels of *GLN2* and *TIP41*, a house-keeping gene, in the shoots of 13-d-old reciprocally-grafted plants between Col (C) and *ami2* (a) 3 d after transfer to media containing 10 mM ammonium (mean ± SD; n = 4 for C/C, C/a, and a/C, n = 3 for a/a). Three shoots from three plates constituted a single biological replicate. Different letters denote significant differences at $P < 0.05$ (Tukey-Kramer's multiple comparison test). NS denotes not significant.

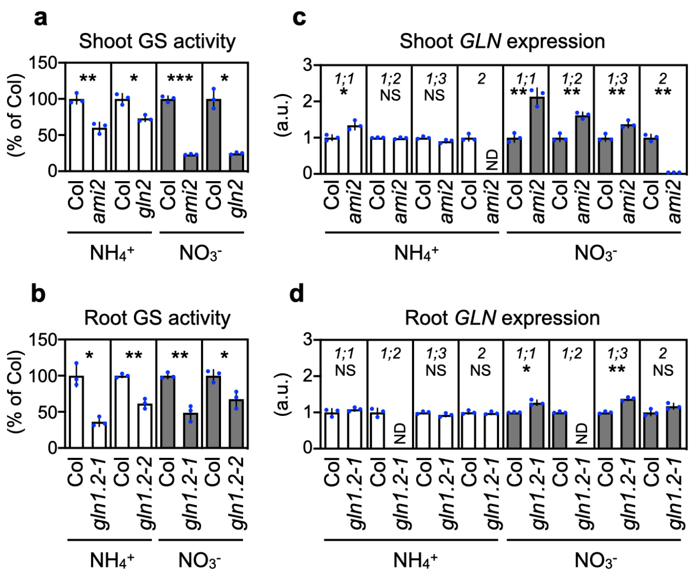

Supplementary Figure 7 Deficiencies in *GLN2* and *GLN1;2* reduce GS activities in shoots and roots, respectively. a Shoot GS activities in 5-d-old Col, *ami2*, and *gln2* grown on media containing 10 mM ammonium or 10 mM nitrate (mean ± SD; n = 3). Thirty-seven shoots from one plate constituted a single biological replicate. **b** Root GS activities in 5-d-old Col, *gln1;2-1*, and *gln1;2-2* grown on media containing 10 mM ammonium or 10 mM nitrate (mean ± SD; n = 3). Thirty-seven shoots from one plate constituted a single biological replicate. **c** Relative transcript levels of *GLN1;1*, *GLN1;2*, *GLN1;3*, and *GLN2* in the shoots of 5-d-old Col and *ami2* grown on 10 mM ammonium or 10 mM nitrate (mean ± SD; n = 3). Thirty-seven shoots from one plate constituted a single biological replicate. **d** Relative transcript levels of *GLN1;1*, *GLN1;2*, *GLN1;3*, and *GLN2* in the roots of 5-d-old Col and *gln1;2-1* grown on media containing 10 mM ammonium or 10 mM nitrate (mean ± SD; n = 3). Thirty-seven shoots from one plate constituted a single biological replicate. **a-d** **P* < 0.05; ***P* < 0.01; ****P* < 0.001 (Welch's *t*-test). NS denotes not significant.

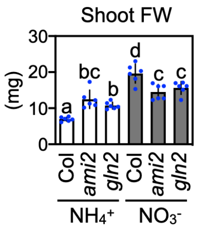

Supplementary Figure 8 *GLN2* deficiency enhances ammonium insensitivity. FW of shoots from 12-d-old Col, *ami2*, and *gln2* 5 d after transfer to media containing 10 mM ammonium or 10 mM nitrate (mean ± SD; n = 6). Three shoots from one plate constituted a single biological replicate. Different letters denote significant differences at P < 0.05 (Tukey-Kramer's multiple comparison test).

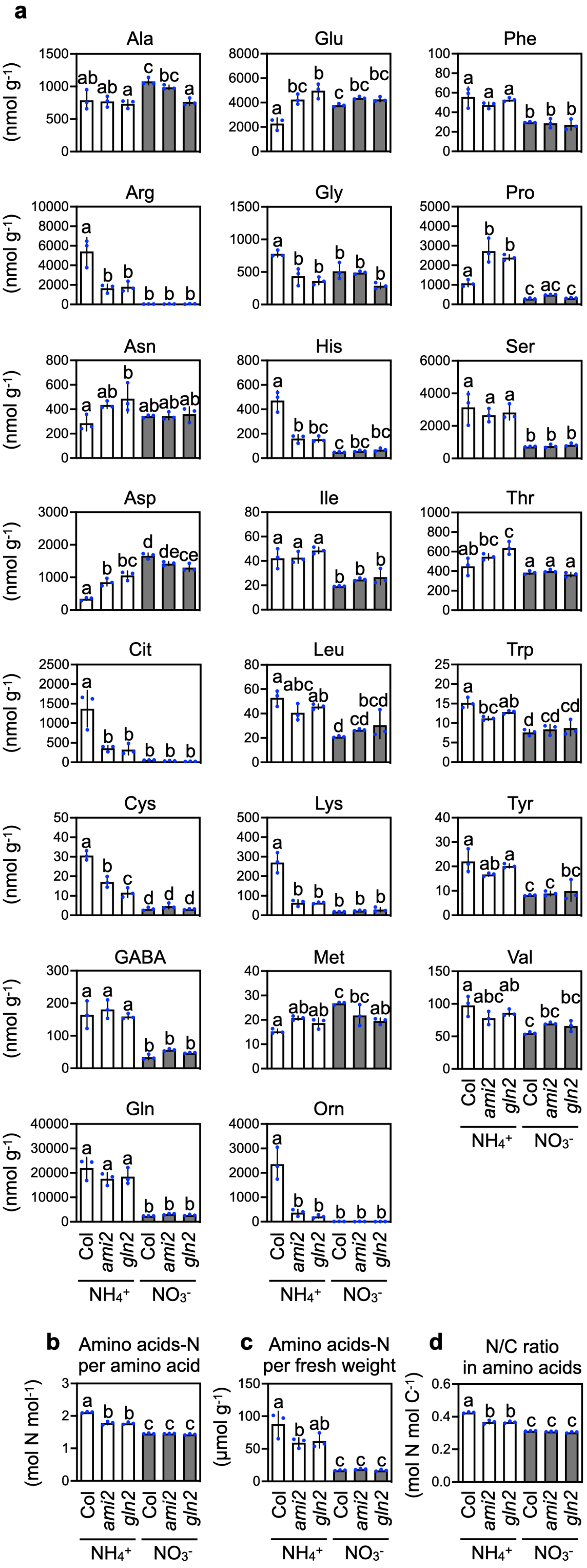

Supplementary Figure 9 *GLN2* deficiency reduces ammonium-N incorporation into amino acids when plants are grown on ammonium. a Amino acid content, b total amino acid-N concentration per amino acid (mol N mol^{-1}), c total amino acid-N concentration per fresh weight (μmol g^{-1}), and d molar ratios of N to C in total amino acids (mol N mol C^{-1}) in the shoots of 12-d-old Col, *ami2*, and *gln2* 5 d after transfer to media containing 10 mM ammonium or 10 mM nitrate. Six shoots from two independent plates constitute one biological replicate. Three biological replicates were sampled separately three times (mean ± SD; n = 3). Different letters denote significant differences at P < 0.05 (Tukey-Kramer's multiple comparison test).

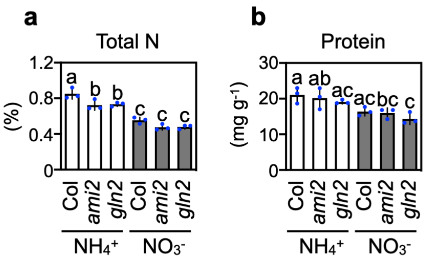

Supplementary Figure 10 *GLN2* deficiency reduces levels of total N and protein when plants are grown on ammonium. **a** Total N concentration (% of fresh weight) and **b** total protein concentration in the shoots of 12-d-old Col, *ami2*, and *gln2* 5 d after transfer to media containing 10 mM ammonium or 10 mM nitrate. Six shoots from two independent plates constitute one biological replicate. Three biological replicates were sampled separately three times (mean ± SD; n = 3). Different letters denote significant differences at $P < 0.05$ (Tukey-Kramer's multiple comparison test).

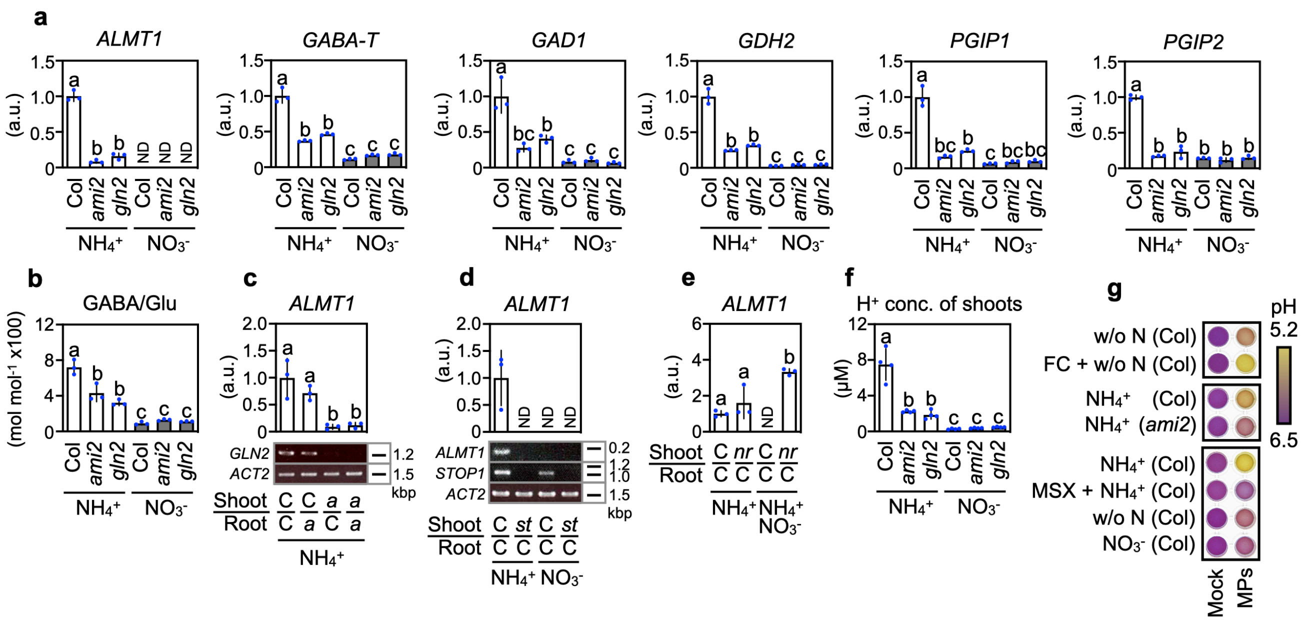

Supplementary Figure 11 Ammonium assimilation by *GLN2* causes acidic stress. a Relative transcript levels of acidic stress-inducible genes[24,25] in the shoots of 10-d-old Col, *ami2*, and *gln2* 3 d after transfer to media containing 10 mM ammonium or 10 mM nitrate (mean ± SD; n = 3). Three shoots from one plate constituted a single biological replicate. **b** The molar ratio of GABA to Glu in the shoots of 12-d-old Col, *ami2*, and *gln2* 5 d after transfer to media containing 10 mM ammonium or 10 mM nitrate (mean ± SE; n = 3). Six shoots from two independent plates constitute one biological replicate. Three biological replicates were sampled separately three times. **c** Relative transcript levels of *ALMT1* in the shoots from 13-d-old reciprocally-grafted plants between Col (C) and *ami2* (a) 3 d after transfer to media containing 10 mM ammonium (mean ± SD; n = 3). Three shoots from three plates constituted a single biological replicate. The transcript levels of *GLN2* and *ACTIN2* (*ACT2*) were evaluated by semi-quantitative RT-PCR with agarose gel electrophoresis. **d** Relative transcript levels of *ALMT1* in the shoots from 13-d-old grafted plants between Col (C) and *stop1-KO* (st) 3 d after transfer to media containing 10 mM ammonium or 10 mM nitrate (mean ± SD; n = 7). Two shoots from two plates constituted a single biological replicate. The transcript levels of *ALMT1*, *STOP1*, and *ACTIN2* were evaluated by semi-quantitative RT-PCR with agarose gel electrophoresis. **e** Relative transcript levels of *ALMT1* in the shoots from 13-d-old grafted plants between Col (C) and the *NR*-null mutant (nr) 3 d after transfer to media containing 10 mM ammonium (NH_4^+) or 2.5 mM nitrate and 10 mM ammonium (NH_4^+ NO_3^-) (mean ± SD; n = 3). One shoot from one plate constituted a single biological replicate. **f** Proton concentrations of water extracts of shoots from 12-d-old Col, *ami2*, and *gln2* 5 d after transfer to media containing 10 mM ammonium or 10 mM nitrate (mean ± SD; n = 4). Six shoots from two plates constituted a single biological replicate. **g** Qualitative evaluation of proton efflux from mesophyll protoplasts (MPs) prepared from Col and *ami2* to liquid media containing 10 mM ammonium (NH_4^+), no N (w/o N), or 10 mM nitrate (NO_3^-) in the presence of 0.02% (w/v) bromocresol purple adjusted to pH 6.7. Fusicoccin (FC), an irreversible activator of plasma-membrane H^+-ATPase, was added at a final concentration of 1 M as a control. A representative photograph is shown. **a-f** Different letters denote significant differences at $P < 0.05$ (Tukey-Kramer's multiple comparison test).

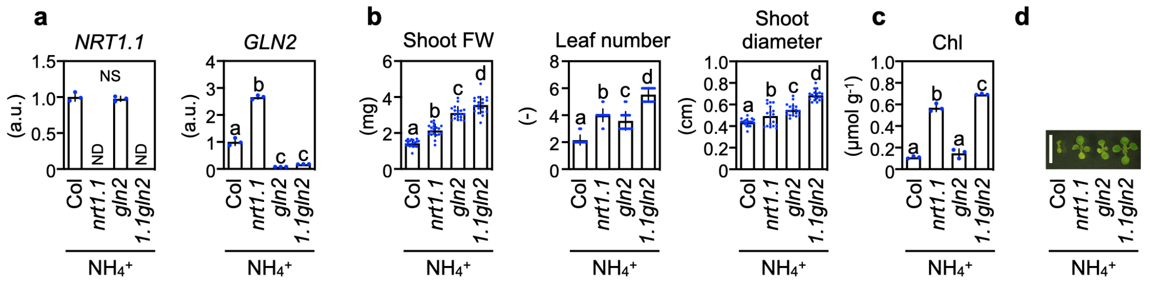

Supplementary Figure 12 Deficiencies in *NRT1.1* and *GLN2* additively enhance ammonium insensitivity. a Relative transcript levels of *NRT1.1* and *GLN2* in seedlings of 5-d-old Col, *nrt1.1*, *gln2*, and *nrt1.1gln2* grown on media containing 10 mM ammonium (mean ± SD; n = 3). Thirty-seven seedlings from one plate constituted a single biological replicate. b Shoot FW, leaf number, and shoot diameter of 11-d-old Col, *nrt1.1*, *gln2*, and *nrt1.1gln2* (mean ± SD; n = 19-20). In one dish, three seeds of each line of Col, *nrt1.1*, *gln2*, and *nrt1.1gln2* were grown on media containing 10 mM ammonium for 11 d. c Chlorophyll content in the shoots of 5-d-old Col, *nrt1.1*, *gln2*, and *nrt1.1gln2* grown on media containing 10 mM ammonium (mean ± SD; n = 3). Thirty-seven shoots constituted a single biological replicate. d A representative photograph of 11-d-old shoots is shown. The scale bar represents 10 mm. a-c Different letters denote significant differences at $P < 0.05$ (Tukey-Kramer's multiple comparison test).

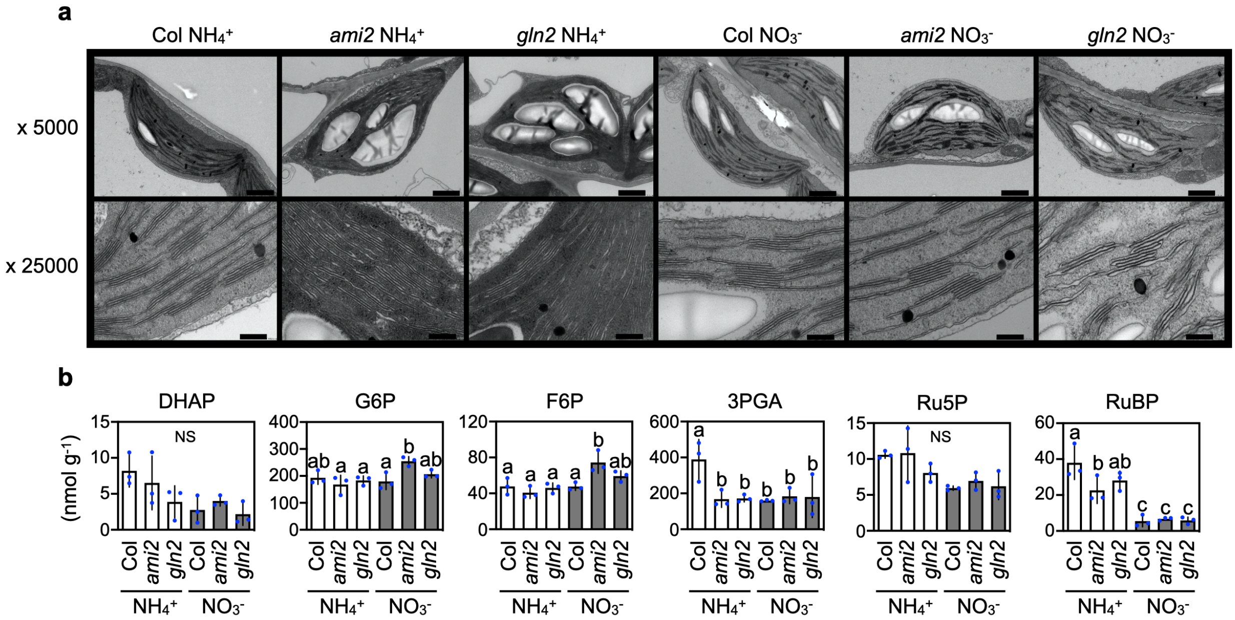

Supplementary Figure 13 Abnormal chloroplast membrane structure and depletion of photosynthetic intermediates were not observed under conditions of ammonium toxicity. a Representative micrographs of chloroplast ultrastructure of mesophyll cells in true leaves of 12-d-old Col, *ami2*, and *gln2* 5 d after transfer to media containing 10 mM ammonium or 10 mM nitrate. Upper and lower panels represent 5,000- and 25,000-fold magnifications, respectively. The scale bars at upper and lower panels represent 1 μm and 200 nm, respectively. b Contents of Calvin-Benson cycle intermediates in shoots of 12-d-old Col, *ami2*, and *gln2* 5 d after transfer to media containing 10 mM ammonium or 10 mM nitrate. Six shoots from two independent plates constitute one biological replicate. Three biological replicates were sampled separately three times (mean ± SD; n = 3). Different letters denote significant differences at $P < 0.05$ (Tukey-Kramer's multiple comparison test).

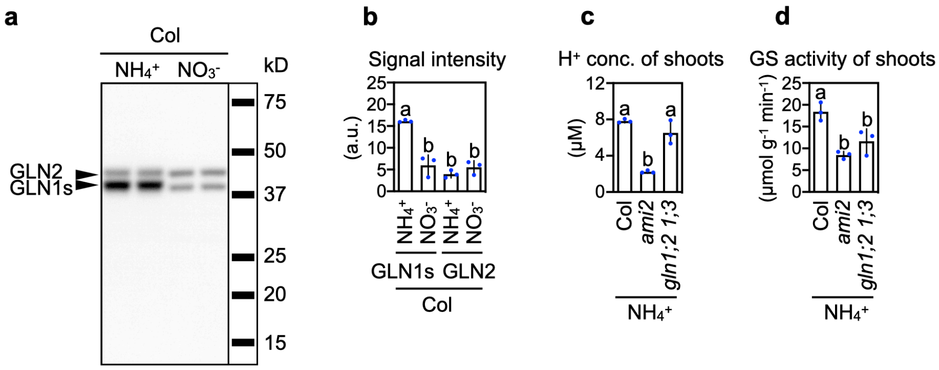

Supplementary Figure 14 The GLN1 protein content in the shoot was higher in plants grown on media containing ammonium than those grown on nitrate-containing media. a Immunodetection of GLN1s and GLN2 isoproteins with specific antisera raised against maize GS following SDS-PAGE and immunoblotting of total protein extracts of 12-d-old Col shoots 5 d after transfer to media containing 10 mM ammonium or 10 mM nitrate. The positions of the molecular weight markers are shown on the right. **b** Signal intensities corresponding to GLN1s and GLN2 isoproteins (mean ± SD; n = 3). Three shoots from one plate constituted a single biological replicate. The intensity was quantified by ImageJ software, version 10.2. **c** Proton concentrations of water extracts of shoots from 12-d-old Col, *ami2*, and *gln1;2 gln1;3* 5 d after transfer to media containing 10 mM ammonium (mean ± SD; n = 3). Two shoots from two plates constituted a single biological replicate. **d** GS activity of shoots from 12-d-old Col, *ami2*, and *gln1;2 gln1;3* 5 d after transfer to media containing 10 mM ammonium (mean ± SD; n = 3). Two shoots from two plates constituted a single biological replicate. **b-d** Different letters denote significant differences at $P < 0.05$ (Tukey-Kramer's multiple comparison test).

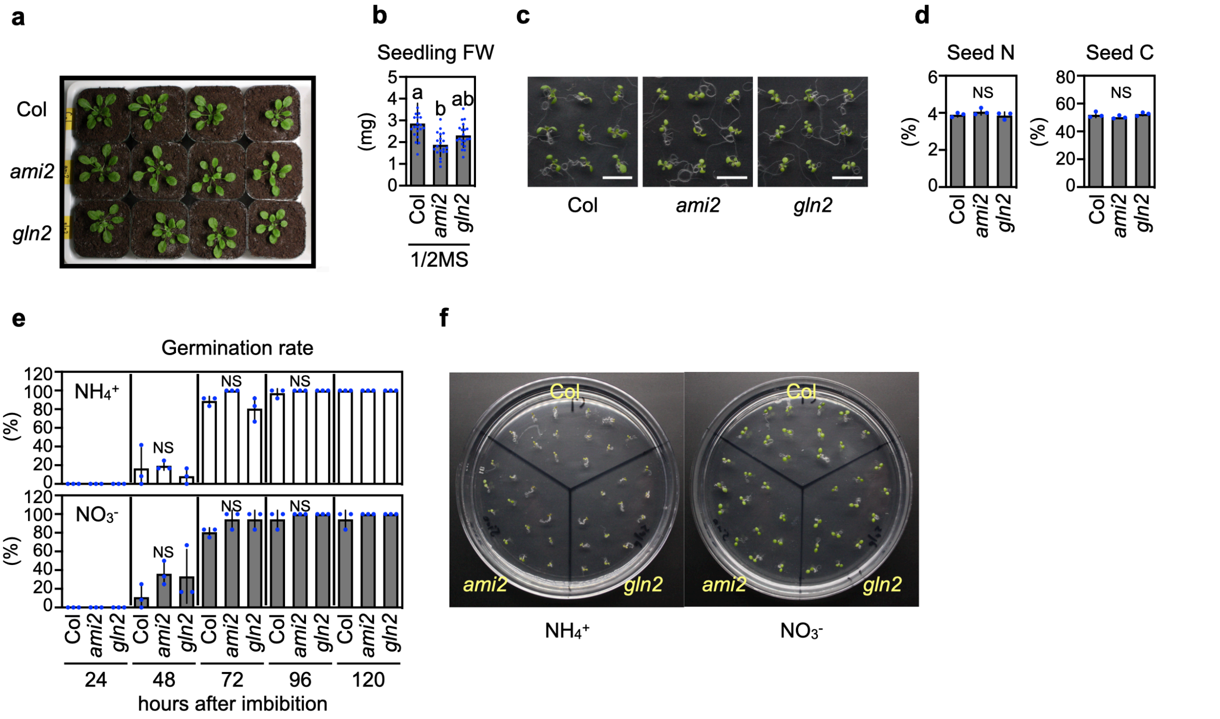

Supplementary Figure 15 Arabidopsis *GLN2*-deficient mutants do not show severe retarded growth under photorespiratory conditions. a A representative photograph of 38-d-old Col, *ami2*, and *gln2* is shown. The plants were pre-cultured for 7 d in half-strength Murashige and Skoog media, transferred to the soil (Sakata Supermix-A, Sakata Seed Corporation, Yokohama, Japan), and grown for further 31 d. **b,c** Fresh weights (FW) and representative photograph of 7-d-old Col, *ami2*, and *gln2* seedlings grown on half-strength Murashige and Skoog media, supplemented with 0.05% (w/v) mM MES, 1% (w/v) sucrose, and 0.25% (w/v) gellan gum (pH 5.7) (mean ± SD; n = 20). **d** Dry weight percent concentratins of total N and C in the seeds of Col, *ami2*, and *gln2*. Three biological replicates were sampled separately three times (mean ± SD; n = 3). **e,f** A time course of germination rates after imbibition of Col, *ami2*, and *gln2* grown on half-strength Murashige and Skoog media, supplemented with 0.05% (w/v) mM MES, 1% (w/v) sucrose, and 0.25% (w/v) gellan gum (pH 5.7). 12 seeds of each line per plate were placed on the media. The germination rate (%) from one plate constitute one biological replicate (mean ± SD; n = 3). Germination was defined as appearance of cotyledon(s). **b,d** Different letters denote significant differences at $P < 0.05$ (Tukey-Kramer's multiple comparison test). NS denotes not significant.
