# Supplementary material for: Excessive ammonium assimilation by plastidic glutamine synthetase causes ammonium toxicity in Arabidopsis thaliana
Source: Nat Commun. 2021 Aug 16;12:4944. doi: 10.1038/s41467-021-25238-7 (PMC8367978; doi:10.1038/s41467-021-25238-7)
Supplement: Supplementary file 3 — Description of Additional Supplementary Files [file 41467_2021_25238_MOESM3_ESM.docx]

**Description of Additional Supplementary Files**

**Supplementary Data 1:** The list of ammonium-inducible genes and ammoniumrepressive genes in A. thaliana shoots

**Supplementary Data 2:** The list of acidic stress-inducible genes and acidic stressrepressive genes in A. thaliana shoots

**Supplementary Data 3:** Primer sequences

**Supplementary Data 4:** Microarray results
